# Diagnostics of *^ν^*_La.max_ and Glycolytic Energy Contribution Indicate Individual Characteristics of Anaerobic Glycolytic Energy Metabolism Contributing to Rowing Performance

**DOI:** 10.3390/metabo13030317

**Published:** 2023-02-21

**Authors:** Frederik Schünemann, So-Young Park, Corinna Wawer, Christian Theis, Woo-Hwi Yang, Sebastian Gehlert

**Affiliations:** 1 Department for Biosciences of Sports, Institute of Sport Science, University of Hildesheim, 31139 Hildesheim, Germany; 2 Graduate School of Sports Medicine, CHA University, Seongnam-si 13503, Republic of Korea; 3 Olympic Training Center Hamburg/Schleswig Holstein, 22049 Hamburg, Germany; 4 Center for Anaesthesiology, Helios University Hospital Wuppertal, 42283 Wuppertal, Germany; 5 Department of Medicine, General Graduate School, CHA University, Seongnam-si 13503, Republic of Korea; 6 Institute of Cardiovascular Research and Sports Medicine, German Sport University Cologne, 50933 Cologne, Germany

**Keywords:** ergometer rowing performance, energetic contribution, glycolytic metabolism, maximum rate of lactate accumulation, metabolic flexibility

## Abstract

The diagnostics of anaerobic glycolytic metabolism which play a subordinate role in elite rowing and parameters such as maximum lactate accumulation rate (*^ν^*_La.max_) have thus far not been associated with ergometer rowing performance. The aim of the study was to quantify the glycolytic energy metabolism (*W*_Gly_) during a 2000 m ergometer rowing time trial (RTT) and *^ν^*_La.max_ during a 10 s maximum ergometer rowing sprint test (RST) and to unravel associations between those variables and RTT performance. Combined post-exercise lactate measurements and oxygen uptake after RST and RTT were used to determine *^ν^*_La.max_ and glycolytic energy contribution (*W*_Gly_) in seven male and three female German U 23 national rowers (N = 10, 19.8 ± 0.9 years, 183.2 ± 7.0 cm height, 79.9 ± 13.3 kg body mass, 16.4 ± 5.1 % body fat). *W*_Gly_ during RTT ranged from 7 to 15.5% and *^ν^*_La.max_ between 0.25 and 0.66 mmol∙L^−1^∙s^−1^. *^ν^*_La.max_ correlated with *W*_Gly_ (*p* < 0.05, *r* = 0.74) and the mechanical power output (W) for the first 300 m (300_first_) during RTT (*p* < 0.05, *r* = 0.67). *^ν^*_La.max_ further correlated with ∆300_first−last_ (W) for the first and last 300 m (300_last_) during RTT (*p* < 0.01, *r* = 0.87) and also within the subgroup of male rowers. *^ν^*_La.max_ displays a wide spectrum of individual differences in rowers. Due to this and its correlation to specific phases of RTT, it contributes to an individual energetic performance profile in rowing. Future studies must undermine the role of *^ν^*_La.max_ for exercise performance and whether it serves as a marker that can be specifically targeted for a training-induced increase or decrease.

## 1. Introduction

Rowing is one of the Olympic disciplines with a particular high demand on all metabolic pathways. At the top level, the 2000 m distance for men and women is covered between 335 and 460 s, depending on the classification, i.e., heavyweight or lightweight, boat classification and sex, and conducted with a mean mechanical power output between 450 to 550 W [1,2]. To facilitate the oxidative supply for such high mechanical power outputs, male elite rowers have an absolute maximum oxygen uptake ($\dot{V}$O_2max_) of up to 7 and female elite rowers of up to 4 L∙min^−1^ [3,4,5,6,7], respectively. A high $\dot{V}$O_2max_ is scientifically undisputed for such performances [8,9,10,11]. The contribution of the oxidative metabolism was determined by de Campos Mello and colleagues to have a mean of 87% on the water and 84% on the ergometer [12]. Importantly, post rowing serum lactate concentrations may rise as high as 32 mmol·L^−1^, thus pointing also to a significant contribution of the anaerobic glycolytic energy metabolism [13,14]. So far, several studies investigated the response and the contribution of the anaerobic glycolytic metabolism in rowing [14,15,16]. De Campos Mello and co-authors determined an anaerobic lactic energy contribution of just 6% on the water and 9% on the rowing ergometer for 2000 m of rowing.

The contribution of anaerobic metabolism to rowing performance is generally more difficult to quantify [16] but was recently redetermined by an analysis of energetic contributions in rowers [17]. Diry et al. calculated an anaerobic glycolytic energy contribution to be a mean of 11.5% for the 2000 m rowing time trial. The anaerobic energy metabolism consists of the phosphagen system fueled by phosphocreatine (PCr) and adenosine triphosphate (ATP) and the glycolytic pathway producing pyruvate or lactate [18,19].

The glycolytic energy metabolism produces considerable amounts of energy per second [20,21,22], especially at workloads above the maximum lactate steady state (MLSS) or the power output above the anaerobic lactate threshold [23]. Although the term and the physiology behind such thresholds are still discussed [24], they nevertheless represent parameters that are frequently used in performance diagnostics. As such, it is used as a rough estimate of the MLSS and the mechanical power output above which lactate production exceeds its oxidation [25].

The 2000 m rowing race consists of specific race phases contributing to the mean mechanical power output and the entire race time [2,26,27,28,29]. As such, the approximate first and last minute are characterized as either phases of acceleration from the start or a sprint up to the finish [2,26,27,28,29]. During these phases, but also in the mid phase of the race, the required mechanical power output outranges the mechanical power output at 4 mmol·L^−1^ (P4) and is around or above the $\dot{V}$O_2max_ [7]. Under such conditions, glycolytic energy metabolism must support the total energy metabolism to fuel the required metabolical power output [14,15,16]. Already in 1984, Heck et al. [30] assumed, that the maximum rate of lactate accumulation (*^ν^*_La.max_), determined via specific sprint tests, may serve as a measure for the individual rate of anaerobic glycolytic energy metabolism in an athlete [20]. Based on mathematical modelling [31] it was shown, that the individual *^ν^*_La.max_ significantly affects the power output at the anaerobic lactate threshold [20]. While a low *^ν^*_La.max_ shifts the lactate curve to the right and improves mechanical power output at the lactate threshold, a high *^ν^*_La.max_ decreases lactate threshold performance [25]. However, particularly high mechanical power outputs, e.g., required in 1000 m time trial in track cycling, are only possible with the support of a high *^ν^*_La.max_ [25].

Performance diagnostics for rowers in Germany usually focuses on the determination of the P4 [30] which is useful to diagnose the individual training progress and to control zones of training intensity. Unfortunately, the role of the glycolytic energy metabolism for rowing performance is still not clear and not mirrored by P4 alone.

Because the 2000 m rowing time trial (RTT) puts high demands on all energy metabolism pathways and the prominent diagnostic procedures in rowing do not specifically test for the glycolytic energy metabolism, the aim of our approach was firstly to determine the contribution of glycolytic energy metabolism during 2000 m RTT performance. Using a mathematical model involving $\dot{V}$O_2_ and lactate accumulation (La^−^) obtained during and post 2000 m RTT, three energy system contributions (anaerobic alactic, anaerobic glycolytic, and oxidative) were calculated in German U 23 national rowers. Furthermore, *^ν^*_La.max_ in a 10 s maximum sprint test (RST) was determined to get an estimate for the interindividual variability of this diagnostic marker in rowers. By correlating *^ν^*_La.max_ with specific phases of 2000 m RTT and the glycolytic energy contribution, we aimed to determine and quantify possible associations that may indicate a specific role of *^ν^*_La.max_ to support energy metabolism during 2000 m RTT and the share of glycolytic energy contribution during 2000 m RTT.

## 2. Materials and Methods

### 2.1. Participants

The sample size was calculated using the G*Power software (G*Power software 3.1.9.4; Heinrich Heine University, Düsseldorf, Germany): effect size = 1.80, alpha error probability = 0.05, statistical power = 0.80. The consideration of effect size was based on previous studies [32,33,34]. Therefore, seven male and three female German U 23 national rowers were recruited, including 20% dropout rate and participated in the study (N = 10). All rowers participated in national and international championships at the time of the study. All tests were conducted in October 2021. Six athletes were heavyweight and four were lightweight rowers. At the time of the study, all athletes were carrying out the general preparation phase for the regular season. Body fat and weight were determined using a Medical Body Composition Analyzer from SECA (SECA GmbH & Co KG, Hamburg, Germany). Body height (cm) was measured electrically with a stadiometer (SECA GmbH & Co. KG, Hamburg, Germany). Data are shown in Table 1.

All participants received written athlete information in advance and were informed about the aim, procedure, and risks of the study. This participant information also contained instructions on which aspects the participants must consider before their individual test days. Some days before the testing procedures, all participants were informed orally and in writing in advance of the study for a second time and gave their written informed consent prior participation. The study was approved by the Institutional Review Board of University of Hildesheim (No. 259_22) and followed the Declaration of Helsinki. The athletes did not perform any strength training or physically demanding rowing training for 48 h before the tests and participated in the rested state.

### 2.2. Lactate Diagnostics

For the determination of lactate concentration, capillary blood samples (mmol·L^−1^, 20 μL) were collected into an end-to-end capillary (EKF diagnostic, Barleben, Germany) from the earlobe. Lactate samples were analyzed immediately after collection using an enzymatic–amperometric sensor chip system (Biosen C-line, EKF diagnostics sales GmbH, Barleben, Germany). An automatic calibration of the system was performed after 60 min. For each test, resting lactate values were ≤2.0 mmol·L^−1^.

### 2.3. Cardio Pulmonary Exercise Testing (CPET)

Oxygen uptake ($\dot{V}$O_2_) was measured using a spirometry system K5 (COSMED, Rome, Italy) in breath by breath mode. The values were averaged at 5 s intervals using Omnia Cardiopulmonary Diagnostic Software version 1.6.10 (COSMED, Rome, Italy). The flow sensor was calibrated with 5 strokes of a high precision 3 L syringe (Hans Rudolph, Kansas City, MO, USA). The gas calibration was performed with calibration gas (16% O_2_ and 5% CO_2_, COSMED, Rome, Italy). All athletes wore silicone masks in sizes M and L which were attached to a flowmeter of the K5 system (Hans Rudolph, Kansas City, MO, USA).

### 2.4. Rowing Ergometers

An air-braked Concept II Type C ergometer was used in each of the tests (Concept II, Morrisville, VT, USA). The sprint tests were conducted on a modified ergometer which was additionally equipped with a FES (Institute for Research and Development, Berlin, Germany) measurement system. This system consists of a load cell for force measurement and a rotary transducer to calculate the power output. Values were submitted to the associated computer software (FES Ruderergo-Dyno v. 2013.9.1, Institute for Research and Development, Berlin, Germany). In the starting position before each test, athletes sat with the rolling seat close to the resting flywheel, i.e., the participants’ knees were in flexion. This position was chosen in each test because it equals the start in the boat. Drag factor settings in the tests were based on the conditions that apply at national Olympic Training Centers and German Rowing Association (DRV). The drag factor on an air-braked rowing ergometer simulates the water resistance that the paddle of the rower must overcome in the boat. A high drag factor in the sprint test was necessary to provide adequate starting resistance for rowers to generate a maximum mechanical power output from the starting position.

### 2.5. Heart Rate

Heart rate (bpm) was measured beat to beat continuously during each test using a Suunto Dual heart rate belt (Suunto, Vantaa, Finland).

### 2.6. Laboratory Conditions

All tests were conducted at a relative humidity of 39–48% and a temperature of 20.3–22.5° Celsius. The values were measured using a Kestrel anemometer (Kestrel Weather and Environmental Meter, Boothwyn, USA).

### 2.7. The 10 s RST

The 10 s RST was conducted on the Concept II Type C rowing ergometer equipped with the FES measurement system, because an external measurement of mechanical power output is imperative when utilizing the C2 for short tests, due to a substantial underestimation of the start strokes [35]. After the warm-up, capillary blood samples were obtained to determine the resting lactate level (La_rest_). In case, the resting lactate values were higher than 2.0 mmol·L^−1^, participants were advised to continue rowing with a low intensity until blood lactate levels were below 2.0 mmol·L^−1^.

The intensity of the warm-up program was kept low (100 W) to avoid effects on maximum post-exercise lactate. Participants conducted the 10 s RST with the highest possible frequency to achieve a maximum mechanical power output during the test. The drag factor was 185 for men and 170 for women. The coaches verbally motivated all participants during 10 s RST for maximal effort. Immediately after the end of the test, the participants remained seated on the ergometer. For the determination of the maximum post-exercise lactate concentration, capillary blood was collected from the earlobe in one-minute intervals over a total time of 10 min. Once the lactate value dropped twice in a row, the test was considered complete and no further blood samples were obtained. The lactate values were needed to calculate the *^ν^*_La.max_.

The formula of *^ν^*_La.max_ is as follows (Equation (1)) [20]:

(1) $${}^{\nu }{}_{\mathrm{La}.\max}\;(\mathrm{mmol}\cdot L^{-1}\cdot s^{-1})=\frac{{\mathrm{La}}_{\mathrm{peak}}-{\;\mathrm{La}}_{\mathrm{rest}}}{t_{\mathrm{Exer}}-{\;t}_{\mathrm{PCr}}}$$

where La_peak_ = the maximum lactate concentration after 10 s RST; La_rest_ = the resting lactate concentration before 10 s RST; t_Exer_ = total exercise (rowing) time (10 s) and t_PCr_ = the time of dominant phosphagen system contribution.

The time of dominant phosphagen system contribution was calculated using an interpolated model that has been reported previously [36] (Equation (2)):


(2) $$t_{PCr}\;(s)=t_{Exer}\cdot 0.0909+2.0455$$

### 2.8. The 2000 m RTT

The 2000 m RTT was performed on the air-braked Concept II Type C rowing ergometer without the FES measurement system because it was not necessary to measure mechanical power output of individual strokes. Before the start, during and after 2000 m RTT, all athletes wore a silicone mask to measure oxygen uptake via CPET. Resting oxygen uptake was measured with each participant seated (5 min). During CPET, two male rowers had to remove their silicone masks because of self-reported breathing problems. However, $\dot{V}$O_2peak_ could be measured for one of these two rowers. For this reason, $\dot{V}$O_2peak_ values could be measured for nine athletes (*n* = 9). Because complete CPET was required for the determination of energy system contributions, these parameters could not be determined for the two athletes mentioned above (*n* = 8). Nevertheless, these two athletes were able to complete the 2000 m RTT. For this reason, the 2000 m performance and the mechanical power output from the first and last 300 m were obtained from all athletes (N = 10).

**Figure 1:**
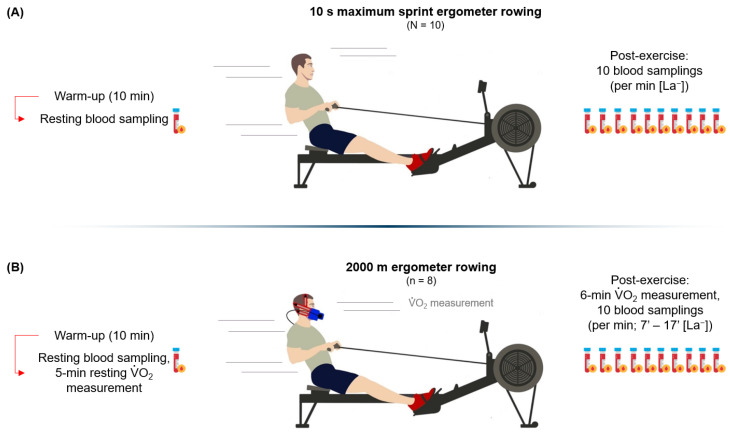
Rowing time trial (RTT) and rowing sprint test (RST) test procedures. (**A**) Procedure of the 10 s RST and (**B**) 2000 m RTT.

All participants were encouraged to run the test with maximum effort, as this is the only way to obtain a reliable and close-to-competition calculation of the energetic contributions. The drag factor was 145 for the men and 130 for the women. After the test, the participants remained seated on the ergometer and $\dot{V}$O_2_ was continuously measured for *off* $\dot{V}$O_2_ kinetics [12] (6 min) (Figure 1B). La_peak_ was determined during post exercise every minute from minute 7 to 17 min. Lactate samples were analyzed immediately after the 2000 m RTT. If two lactate values decreased twice in a row, the blood samplings were considered to complete and no further collections were obtained. The entire test was completed after the last post-exercise lactate collection after reaching La_peak_. The main variables of the 2000 m RTT were:

(1) 2000 m RTT performance (s).
(2) Average power over the first and last 300 m of 2000 m RTT (P300_first_ and P300_last_ in W).
(3) Mechanical power output difference between 300 m first and last (∆300 _first-last_).
(4) $\dot{V}$O_2_ (litres O_2_·min^−1^) before, during, and after 2000 m RTT.
(5) Resting lactate and peak blood lactate concentration after 2000 m RTT.

### 2.9. The Incremental Step Test

The incremental step test was performed on the air-braked Concept II Type C rowing ergometer with FES measurement system. The test consisted of five stages, each with a duration of 4 min and was conducted according to the DRV standards in ergometer rowing. After a low intense warm-up on the rowing ergometer of around 5 min (100 W), capillary blood samples were obtained to determine the resting lactate concentration (La_rest_). For men, the power of the first stage was 150 W. Female participants started at 80. After each stage, there was a half-minute break before the next one followed. During the 30-s rest period, heart rate and blood lactate were measured and the individual rate of perceived exertion (RPE) was queried. Power output was increased by 50 W per stage for men and 40 W for women. Accordingly, the fifth and final stage resulted in 350 W for men and 240 W for women. On a screen, in front of the ergometer, the athletes were able to see whether they met the required mechanical power output. If the average of the last three strokes was 10 W or more below the target power output, athletes received a visual warning that they must increase mechanical power output. After the fifth warning, the test was automatically terminated. This test is only possible with the FES system and the associated software. At the end of the test, post-exercise lactate measurements were conducted at 3, 5, and 7 min. The drag factor was 145 for men and 130 for women. Thus, the P4 on the rowing ergometer was determined.

### 2.10. Calculations of Energetic Contributions during 2000 m RTT

Phosphagen, glycolytic, and oxidative contributions were estimated by the assessment of $\dot{V}$O_2_ (during 2000 m RTT), peak La^−^, and the fast component of excess $\dot{V}$O_2_ after exercise (EPOC_FAST_), respectively [32,33,34,37,38,39]. The phosphagenic energy contribution (*W*_PCr_) was calculated considering $\dot{V}$O_2_ after 2000 m RTT, and the fast component of excess post exercise after 2000 m RTT [34,37,40]. The kinetics of post-exercise $\dot{V}$O_2_ was fitted by mono- and bi-exponential models using OriginPro 2021 (OriginLab Corp, Northampton, Massachusetts, USA) and the slow component of the bi-exponential model was negligible. Therefore, the post-exercise $\dot{V}$O_2_ data were fitted to a mono-exponential model, and *W*_PCr_ was calculated by estimating the integral of the exponential area [12,32,37,39]. The glycolytic energy contribution (*W*_Gly_) was considered by the lactate concentration after 2000 m RTT, assuming that the accumulation of 1 mmol∙L^−1^ (La^−^) is equivalent to 3 mL O_2_∙kg^−1^ of body mass [41]. The delta lactate (ΔLa^−^) was calculated by subtracting the resting lactate concentration from the highest La^−^ after 2000 m RTT [34,37,38]. The oxidative energy contribution (*W*_Oxi_) was calculated by subtracting the resting $\dot{V}$O_2_ ($\dot{V}$O_2rest_) from $\dot{V}$O_2_ levels during 2000 m RTT by the trapezoidal method. In this method, the area under the curve is divided into sections and then the sum of the trapezoid is utilized to calculate the integral [12]. $\dot{V}$O_2rest_ was measured in the sitting position, within the last 30 s of a 5 min period used as a reference value [33,34,37,39]. A caloric quotient of 20.92 kJ was used in all three energetic contributions [18]. Total energetic expenditure (*W*_Total_) was calculated as the sum of the three energy systems (*W*_Oxi_ + *W*_PCr_ + *W*_Gly_) in kJ [32,39]. The relative energy contributions are displayed as percentages (%).

### 2.11. Statistical Analyses

All data were analyzed using GraphPad Prism (version 9.4.1, GraphPad Prism Software Inc., La, Jolla, CA, USA). Parameters are presented as arithmetic mean and standard deviation (SD). The Friedman repeated-measures rank test with Dunn’s post hoc was utilized to compare the energy system contributions during the 2000 m rowing ergometer test (n = 8, in kJ and %). The α-level of significance was set at *p* < 0.05 for all statistical analyses. The effect sizes were calculated for non-parametric tests (ES Kendall’s [W] and $\frac{Z}{\sqrt{N}}$ [r]). The threshold for small, medium, and large effects were considered < 0.3, > 0.3−0.5, and > 0.5 [W] and 0.1, 0.3, and 0.5 [r], respectively [42]. Furthermore, Spearman rank correlation analyses were performed among *W*_Gly_, *^ν^*_La.max_, 2000 m RTT, P300_first_ and P300_last_ of 2000 m RTT, ∆300_first−last_ and P4.

## 3. Results

### 3.1. Calculation of the Three Energy System Contributions (PCr-La^−^-O_2_ Method) during 2000 m RTT

**Figure 2:**
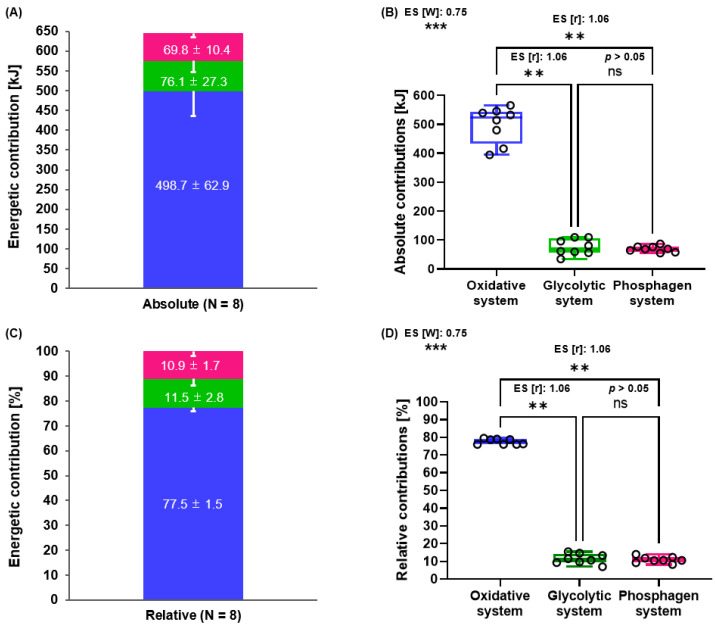
Energetic contributions during 2000 m RTT. Calculated absolute ((**A**,**B**), kJ) and relative ((**C**,**D**), %) energetic contributions in 2000 m RTT. Oxidative energy contribution is shown in blue, glycolytic energy contribution in green, and phosphagen energy contribution in red. Data are mean ± standard deviation (SD) (n = 8). ** *p* < 0.01, *** *p* < 0.001, ns: *p* > 0.05. ES: effect size.

The first aim was to determine the energetic contributions towards 2000 m RTT. Figure 2 displays the three energy system contributions (oxidative, glycolytic, and phosphagen) in kJ (2A,B) and % (2C,D) during 2000 m RTT (*W*_PCr_ in kJ: 69.8 ± 10.4, *W*_Gly_ in kJ: 76.0 ± 27.3, *W*_Oxi_ in kJ: 498.7 ± 62.9, *W*_PCr_ in %: 10.9 ± 1.7, *W*_Gly_ in %: 11.5 ± 2.8, *W*_Oxi_ in %: 77.5 ± 1.5, respectively).

Significant differences in the energetic contributions in kJ and % (*p* = 0.002) were found between those pathways. Values of *W*_Oxi_ in kJ and % were significantly higher compared to *W*_Gly_ and *W*_PCr_ (vs. *W*_Gly_ in kJ; *p* = 0.008, vs. *W*_PCr_ in kJ; *p* = 0.008, vs. *W*_Gly_ in %; *p* = 0.008, vs. *W*_PCr_ in %; *p* = 0.008) (Figure 2B,D). There was neither a significant difference between *W*_Gly_ and *W*_PCr_ in kJ nor in % (*p* > 0.05).

### 3.2. ^ν^_La.max_ and t_PCr_ of 10 s RST, P300_first_, and P300_last_ of 2000 m RTT

Next, the aim was to determine in which range *^ν^*_La.max_ is displayed in the group of participants. *^ν^*_La.max_ values ranged from 0.25 mmol·L^−1^ (P3) as the lowest value to 0.66 mmol·L^−1^ (P6) as the highest value. During the first 300 m, the mean mechanical power output ranged between 466 W (P5) and 218 W (P3). The mechanical power output on the last 300 m to the finish of the 2000 m RTT ranged from 433 W (P8) to 218 W (P3).

### 3.3. The Relationship between ^ν^_La.max_ and W_Gly_ over 2000 m RTT

**Figure 3:**
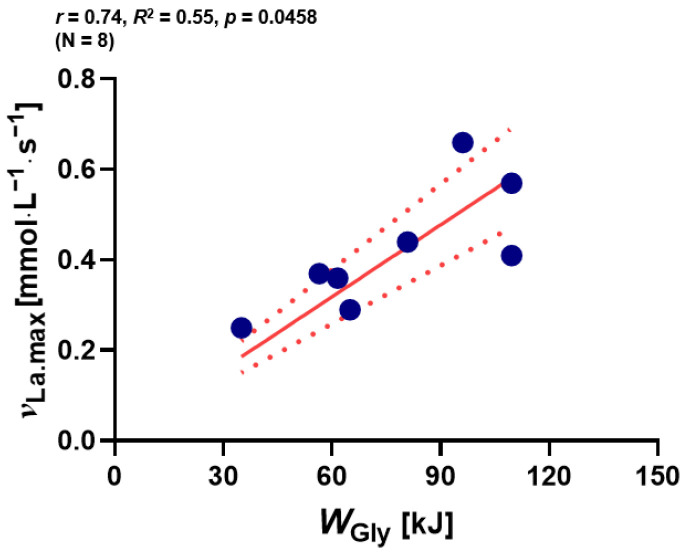
Relationship between ^ν^_La.max_ and *W*_Gly_ (*n* = 8). *^ν^*_La.max_: maximum rate of lactate accumulation, *W*_Gly_: glycolytic energy contribution. Dotted lines: 95% confidence interval, straight line: strength of the linear relationship between two variables.

The next aim was to determine whether ^ν^_La.max_ contributes to the glycolytic energy contribution (in kJ). A positive correlation was observed between W_Gly_ and ^ν^_La.max_ (*p* < 0.05; Figure 3).

### 3.4. The Relationship between ^ν^_La.max_, W_Gly_, P4, Absolute $\dot{V}$O_2peak_, and 2000 m RTT Performance

**Figure 4:**
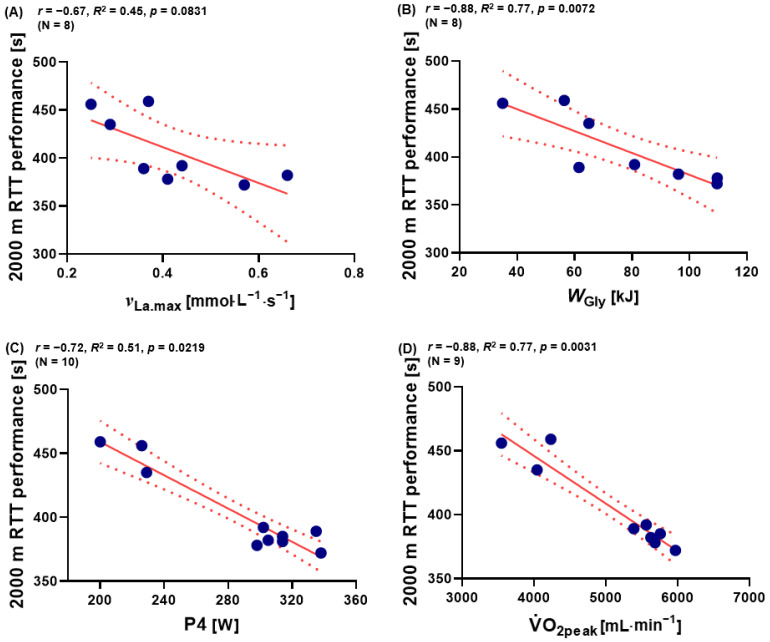
Relationship between (**A**) 2000 m RTT performance and ^ν^_La.max_ (*n* = 8), between (**B**) 2000 m RTT performance and *W*_Gly_ (*n* = 8), between (**C**) P4 and 2000 m RTT performance (*N* = 10), and between (**D**) 2000 m RTT performance and $\dot{V}$O_2peak_ (*n* = 9). $\dot{V}$O_2peak_: peak oxygen uptake during 2000 m RTT, P4: mechanical power output at 4 mmol·L^−1^. Dotted lines: 95% confidence interval, straight line: strength of the linear relationship between two variables.

Based on these findings, it was analyzed whether *^ν^*_La.max_, *W*_Gly_, $\dot{V}$O_2peak_, or P4 has an influence on the 2000 m RTT performance. In addition, significant correlations were seen between *W*_Gly_, P4, $\dot{V}$O_2peak_ and 2000 m RTT performance (*p* < 0.05, *p* < 0.001, and *p* < 0.001). There was no significant correlation between *^ν^*_La.max_ and 2000 m RTT performance for the entire group (*p* > 0.05). (Figure 4A−D).

### 3.5. The Influence of ^ν^_La.max_ on Specific Sections of 2000 m RTT 

**Figure 5:**
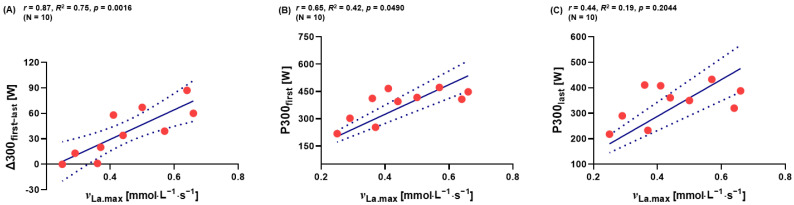
Correlation analyses. Relations between (**A**) the *^ν^*_La.max_ value and ∆300_first−last_ during 2000 m RTT (*N* = 10), between (**B**) first 300 m during 2000 m RTT and *^ν^*_La.max_ (*N* = 10), and between (**C**) last 300 m during 2000 m RTT and *^ν^*_La.max_ (*N* = 10). ∆: Delta 300_first−last_; subtraction of P300_first_ and P300_last_. Dotted lines: 95% confidence interval, straight line: strength of the linear relationship between two variables.

Because mechanical power output over the first and the last 300 m of 2000 m RTT are usually higher than over the middle part of the race, it was investigated whether there was a relationship between the *^ν^*_La.max_ and the delta (∆300_first−last_) value. Similarly, it was analyzed for a correlation between *^ν^*_La.max_ and on the first and last 300 m during 2000 m RTT. A correlation between the *^ν^*_La.max_ value and ∆300_first−last_ was found (*p* < 0.05, Figure 5A). Furthermore, a correlation was found between P300_first_ W during the 2000 m RTT and *^ν^*_La.max_ (*p* < 0.05, Figure 5B). No correlation was found between the P300_last_ W during the 2000 m RTT and *^ν^*_La.max_ (*p* > 0.05, Figure 5C).

### 3.6. Separate Classification of ^ν^_La.max_, $\dot{V}$O_2peak_, and Performance over P300_first_ and ∆300_first−last_ for Male Athletes

**Figure 6:**
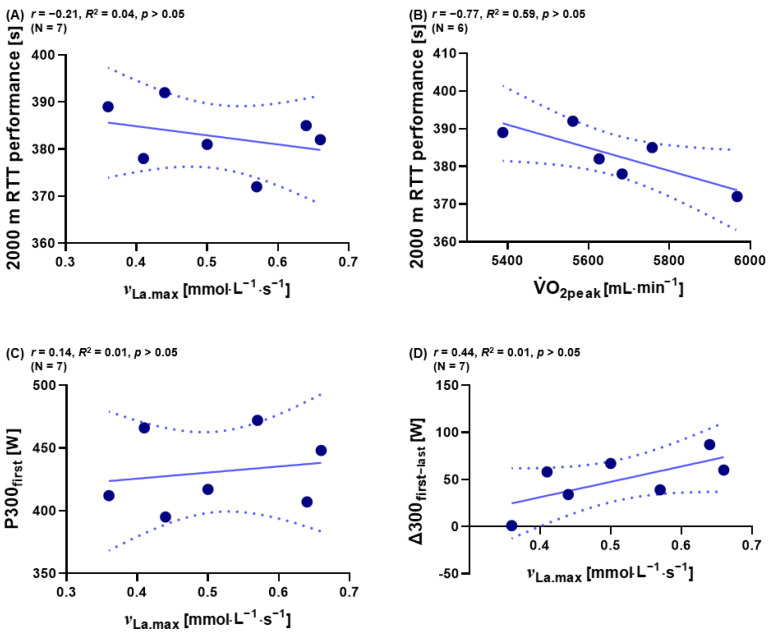
Relations between (**A**) 2000 m RTT performance and *^ν^*_La.max_ (*n* = 7), between (**B**) 2000 m RTT performance and $\dot{V}$O_2peak_ (*n* = 6), (**C**) between P300_fist_ during 2000 m RTT performance and *^ν^*_La.max_ (*n* = 7), and between (**D**) ∆300_first−last_ of the 2000 m RTT and *^ν^*_La.max_ (*n* = 7). Dotted lines: 95% confidence interval, straight line: strength of the linear relationship between two variables.

It was recognized that the obtained correlation between variables in the entire group of rowers was enhanced due to the involvement of three female athletes with generally lower performance values. Those athletes had an overall lower bodyweight, lower muscle mass and therefore $\dot{V}$O_2peak_ and ^ν^_La.max_ what strengthened our interpretation. Therefore, the former correlations for the subgroup of males were reanalyzed in our study. In this subgroup ^ν^_La.max_ and $\dot{V}$O_2peak_ did not correlate with 2000 m RTT performance (*p* > 0.05; *n* = 7; Figure 6A, *p* > 0.05; *n* = 6, Figure 6B). Furthermore, P300_first_ showed no interdependency with ^ν^_La.max_ in the cohort of males (*p* > 0.05; *n* = 7, Figure 6C). The value of ∆300 _first−last_ showed no correlation with ^ν^_La.max_, also in males (*p* > 0.05; n = 7, Figure 6D).

## 4. Discussion

In recent years, the determination of anaerobic metabolism in performance testing [36,43,44,45,46] became more popular. Past [20] and recent findings [25] attribute to the anaerobic glycolytic metabolism a high importance for endurance exercise performance [25]. When exercise is conducted at mechanical power outputs at and above the anaerobic threshold or $\dot{V}$O_2max_ [25] the contribution of the anaerobic glycolytic metabolism more and more increases. Such conditions are predominant during the 2000 m RTT in rowing [14,15,25]. Therefore, the anaerobic glycolytic metabolism can be considered as a factor that enables the high energy flow rate required during rowing.

Because approaches to quantify anaerobic glycolytic performance in rowing diagnostics are still missing, *^ν^*_La.max_ as a measure of one’s individual lactate accumulation rate or anaerobic glycolytic performance in U 23 national rowers was determined in an explorative manner. We further analyzed the contribution of anaerobic glycolytic metabolism during 2000 m RTT and calculated associations between 2000 m RTT performance and *^ν^*_La.max_.

Our calculations revealed a mean contribution of 11.5% of anaerobic glycolytic metabolism during 2000 m RTT in our participants. The shorter the time course in which a maximum mechanical power output has to be maintained, the higher it is [47]. As a consequence, the percentage of anaerobic glycolytic metabolism is also higher. Thus, participants with a shorter race time had a higher percentage of anaerobic glycolytic energy contribution (Figure 4B). The international rowing federation (World Rowing) established the reduction in the course length from 2000 m to 1500 m for men and women for the Olympic Games in 2028 [48]. This will likely increase the absolute and relative anaerobic lactic energy contribution for the competing athlete during a 1500 m race and might affect the training preparation after 2024. A recent study [17] already calculated the changing metabolic demands for this reduction in course length and described an increase for the mean percentage of anaerobic glycolytic energy metabolism from 11.5% to 13.3%.

Our calculation revealed a percentage of 11.5 ± 2.9% which corresponds with the data from Diry and colleagues [17]. The used method is established to determine the proportions of the energy metabolism towards exercise [33,37,49,50,51,52,53]. However, our study showed a higher phosphagen contribution (10.9%) than the results from Diry et al. [17] (1.5%) because of the different methodological calculations of the phosphagen system. The time constant of mono-exponential function using *off* $\dot{V}$O_2_ kinetics, which indicates phosphocreatine (PCr) recovery (return to baseline PCr levels) after intense exercise, serves as a more precise method to consider the phosphagen contribution in our study [33,37,52,53,54].

The *^ν^*_La.max_ during 10 s RST was determined to analyze in which range this easily detectable marker of anaerobic glycolytic energy metabolism displays itself in our group of participants and whether it is related to the glycolytic energy contribution over 2000 m RTT. Within the tested athletes, *^ν^*_La.max_ values between 0.25 and 0.66 mmol·L^−1^·s^−1^, $\dot{V}$O_2peak_ values between 3.5 and 5.9 L·min^−1^, and P4 values between 200 and 338 W were observed (Table 2 and Table 3).

This reveals a broad spectrum of differential energetic abilities in national German U 23 rowers. Importantly, *^ν^*_La.max_ varied between the athletes more than 2-fold while $\dot{V}$O_2peak_ values varied by only 1.7-fold. Thus, *^ν^*_La.max_ displays itself as a diagnostic parameter that reveals a larger range of *^ν^*_La.max_ than $\dot{V}$O_2peak_ in rowers. *^ν^*_La.max_ influences the power at the MLSS and by that also the lactate threshold [25]. Ingham and colleagues have determined that mechanical power output at 2 mmol·L^−1^ and at 4 mmol·L^−1^ is a strong predictor of 2000 m rowing performance [2]. Therefore, the influence of *^ν^*_La.max_ on rowing performance is comprehensible. It was unexpected that *^ν^*_La.max_ differs considerably between athletes otherwise having a similar $\dot{V}$O_2peak_ and 2000 m RTT performance (Table 2 and Table 3, participants P6 and P7).

Importantly, as expected, absolute $\dot{V}$O_2peak_ correlated well with 2000 m RTT performance (Figure 4D). However, although the importance for possessing a high oxidative capacity in rowing is undisputed, the entire performance over 2000 m RTT is seemingly also influenced by anaerobic glycolytic performance as measured by *^ν^*_La.max_.

In the entire group of participants (males and females combined), a correlation was found between *W*_Gly_ during 2000 m RTT and *^ν^*_La.max_ (Figure 3). This indicates that *^ν^*_La.max_ may support the glycolytic flux during exercise and by that the utilization of anaerobic glycolytic energy metabolism. In addition, a correlation was found between *^ν^*_La.max_ and the mechanical power output generated during the first 300 m in the 2000 m RTT (Figure 5A) emphasizing that the individual characteristic of mechanical power output during rowing in this time frame is influenced by the individual glycolytic capacity.

Interestingly, the decline in mechanical power output between the first and last 300 m during 2000 m RTT also correlated with *^ν^*_La.max_. This may attribute *^ν^*_La.max_ a contributing role towards fatigue during specific phases of 2000 m RTT, because athletes with a higher *^ν^*_La.max_ also show a higher reduction in the possible mechanical power output at the end of 2000 m RTT.

Our data imply that an extended performance diagnostic in rowing, including *^ν^*_La.max_ testing, may be feasible to assess one’s individual ability for glycolytic energy metabolism via a non-complicated and time-efficient test procedure.

The concept of *^ν^*_La.max_ as a measure of the individual maximum lactate accumulation rate was introduced by Mader in the 1980s [31]. Approaches to determine *^ν^*_La.max_ so far are used for performance diagnostics in running [55] and cycling [56]. A key determinant for such testing is on the one hand to induce a rapid and maximum turnover of ATP and PCr in order to maximally activate glycolysis [20]. On the other hand, the time frame of the test has to be as short as possible to minimize the activation of oxidative metabolism and thus lactate oxidation [20]. Further, this aims to prevent the increasing inhibition of phosphofructokinase due to declining pH levels [57]. In such tests, the maximum accumulated lactate in blood serves here as an estimate of what working muscle has produced during 10 s maximum RST.

We chose 10 s for the RST, a duration which has been described to minimize oxidative metabolism but is sufficient to maximally activate glycolysis [20]. To calculate *^ν^*_La.max_, we used a phosphagen-contributed time frame of 2.95 s in which energy metabolism is fueled by high energy phosphates and glycolysis is not yet maximally activated and followed already published calculations [36]. While sprint tests designed to determine *^ν^*_La.max_ in running and cycling are described to last between 15 and 30 s [36,46,58,59,60] and allow for more than 50 maximum muscle contractions, rowing is associated with a much slower movement speed during each rowing cycle. Hence, during RST our participants were merely able to conduct between 8 and 11 rowing cycles within 10 s (data not shown). Therefore, due to the reduced number of muscle contractions during RST when compared to cycling maximum lactate values might be lower in our study. Overall, 10 s of RST is technically demanding why participants were able to practice the starting phases in advance of the testing day. It has to be mentioned that individual strength abilities may interfere with the ability to quickly generate force from the start. This can be specifically different between male and female athletes.

Importantly, three of the participants were female and those were the athletes in our correlations (Figure 4B,D) with the lowest $\dot{V}$O_2peak_, but also low *^ν^*_La.max_ values (Table 2 and Table 3, participants P1–P3). Female athletes have generally a lower amount of muscle mass and mean mechanical power output than compared to men [61] which consequently led to an extended competition time of 450 s in women compared to 382.7 s in men in our study. The lower muscle mass also reduces the absolute oxidative capacity due to an absolute lower content of oxidative enzymes.

Due to the mix of female and male athletes, our correlations display a broad range of *^ν^*_La.max_, $\dot{V}$O_2peak_, and P4 values. This range likely made the detection of our findings possible and enhanced our correlations as presented here. In the subgroup of men, our associations (Figure 6A−D) that could be seen in the mixed group of participants could no longer be revealed. All seven men showed a very high 2000 m RTT performance with a *^ν^*_La.max_ variation between 0.36 and 0.66 mmol·L^−1^ and only a weak correlation was found between 300_first_ and 300_last_ but also no correlation was found with $\dot{V}$O_2peak_ and 2000 m RTT performance.

This indicates that our sample size was too small for detecting clear relations in men and based on our findings for the entire group of participants, there is the need for the evaluation of diagnostic markers of energy metabolism and especially *^ν^*_La.max_ in a more extended sample size of elite, sub-elite but also novice rowers. This, in order to clearly estimate the influence of *^ν^*_La.max_ for rowing performance and further to determine under which condition and for which athlete-specific training might be used to increase or decrease *^ν^*_La.max_.

As recently introduced by Wackerhage and colleagues [25], the influence of *^ν^*_La.max_ on exercise metabolism and endurance performance can be interpreted only in combination with $\dot{V}$O_2peak_. As indicated in Table 3 and Figure 6D, the reduction in mechanical power output between the first and last 300 m may indicate a performance decrement which occurs under the influence of *^ν^*_La.max_. Whether either an increase or a decrease in *^ν^*_La.max_ would be an important training goal to enhance 2000 m or 1500 m RTT cannot be determined in our small group of participants and in this initial approach.

To date, there are also no clear data available how to specifically modulate individual *^ν^*_La.max_ by training. Also, *^ν^*_La.max_ itself is yet not a specific topic themed on a regular basis in international exercise physiology. Therefore, literature for this very context is still sparse. Data suggest, that glycolytic capacity will be reduced when oxidative capacity increases [62]. Mechanistically, increasing volumes of training are associated with an augmented expression of PGC-1 alpha-regulated oxidative genes concomitantly with a reduction in glycolytic gene expression [63].

Additionally, a higher potential to oxidize lactate in mitochondria will then reduce blood lactate levels at a given rate of glycolysis, even when the glycolytic potential per se may be increased. This would limit the achievability and detectability for a desired increase in *^ν^*_La.max_ during specific phases of training and in elite athletes who train generally with high training volumes. Several studies show that sprint training may increase the expression of anaerobic glycolytic enzymes or anaerobic performance [64] while others reported no increase in glycolytic capacity after explosive strength training [65,66]. Lactate production also depends on the amount of fast fiber types [67] in skeletal muscle as well as muscle mass [68] which can be increased by resistance training. In this context, resistance training has been shown to increase particularly the hypertrophy and abundance of intermediate fast glycolytic type IIa fibers [69]. In contrast, endurance training does not induce substantial hypertrophy but can induce the transition of myofibers to a slower phenotype, especially when athletes possess a higher amount of fast fibers [70].

Therefore, some approaches showed that resistance training was able to increase *^ν^*_La.max_ [71] and that a reduction in training volume may enhance glycolytic capacity and anaerobic performance [72]. Additionally, hypoxic training methods such as intermittent hypoxic training (IHT) can be effective training strategies to increase anaerobic performance [73]. It has been shown that hypoxia increases the HIF-1 alpha-driven expression of glycolytic enzymes [74].

Based on the former considerations and the high training volumes conducted in rowing, a directed and training-induced increase in *^ν^*_La.max_ may be more difficult to reach than its reduction. Future studies must show under which conditions (e.g., $\dot{V}$O_2peak_) an increase or decrease in *^ν^*_La.max_ may increase rowing-specific performance. Specific training regimens could then be incorporated into rowing training to modulate *^ν^*_La.max_.

### Limitations

Despite our group of highly trained German U23 national rowers, the sample size is still limited. A broader spectrum of 2000 m RTT performances as well as *^ν^*_La.max_, $\dot{V}$O_2peak_, and segments of rowing-specific performance would have greatly undermined our findings and more precisely enclose our interpretations in more extended and significant correlations. Out of 10 participants, only eight data sets could be used for the determination of energy turnover during 2000 m RTT and three of our data sets were from female athletes which also determined the lower limit in our correlations concerning *^ν^*_La.max_, rowing performance during 2000 m RTT, and P300_first_. Hence, those data have influenced our correlations. Although energetic contributions in female athletes follow generally the same physiological regulations as in men, specificities for female athletes exist [61] that must be separately addressed in the future. 

## 5. Conclusions

Although energy supply during 2000 m RTT is predominantly ensured by the oxidative metabolism, the anaerobic glycolytic metabolism, as determined by *^ν^*_La.max_, arises as an individual component of energetic performance that supports the total energy requirements of 2000 m RTT. Based on its broad variety in athletes and its relationship to specific phases of 2000 m RTT, it may serve in the future as a diagnostic marker in rowing to detect potential limiting factors of exercise performance beyond usual diagnostic markers.

## Figures and Tables

**Table 1:** Anthropometric data.

Parameters	Total	Male	Female
	*N* = 10	*n* = 7	*n* = 3
Age [years]	19.80 ± 0.9	19.8 ± 0.9	19.6 ± 0.5
Height [cm]	183.20 ± 7.0	188.4 ± 3.2	175.6 ± 4.7
Body mass [kg]	79.9 ± 13.3	85.4 ± 11.2	67.2 ± 3.8
Body fat [%]	16.4 ± 5.1	14.8 ± 5.0	20.08 ± 3.1

Data are presented as means and standard deviation (SD).

**Table 2:** Physiological variables obtained during and after 2000 m RTT and power output (W) at 4 mmol∙L^−1^ determined during the incremental step test.

Participants (Sex)	2000 m RTT Performance	La_peak_	$\dot{V}$O_2peak_	P4
	s	mmol·L^−1^	ml·min^−1^	W
P1 (f)	435	15.77	4042	229
P2 (f)	459	15.77	4233	200
P3 (f)	456	8.95	3546	226
P4 (m)	392	18.82	5561	302
P5 (m)	378	21.30	5683	298
P6 (m)	382	19.66	5626	305
P7 (m)	389	15.01	5388	335
P8 (m)	372	18.02	5967	338
P9 (m)	385	13.51	5757	314
P10 (m)	381	15.91	-	314

2000 m RTT performance (s), peak lactate values after 2000 m RTT (La_peak_), $\dot{V}$O_2peak_ values (in ml·min^−1^) during 2000 m RTT and mechanical power output at 4 mmol∙L^−1^ (in W) for each athlete (P1–P10).

**Table 3:** Maximum rate of lactate accumulation (^ν^_La.max_) and power output (W) over certain sections during 2000 m RTT.

Participants (Sex)	*^ν^*_La.max_	t_PCr_	P300_first_	P300_last_	∆300 _first-last_
	mmol·L^−1^·s^−1^	s	W	W	W
P1 (f)	0.29	2.95	303	290	13
P2 (f)	0.37	2.95	253	233	20
P3 (f)	0.25	2.95	218	218	0
P4 (m)	0.44	2.95	395	361	34
P5 (m)	0.41	2.95	466	408	58
P6 (m)	0.66	2.95	448	388	60
P7 (m)	0.36	2.95	412	411	1
P8 (m)	0.57	2.95	472	433	39
P9 (m)	0.64	2.95	407	320	87
P10 (m)	0.50	2.95	417	350	67

*^ν^*_La.max_ values (in mmol·L^−1^·s^−1^), t_PCr_ (s), mean mechanical power output (in W) over the first and last 300 m over the course of 2000 m RTT and ∆300 _first-last_ for each athlete (P1−P10).

## Data Availability

Data available on request are dependent on restrictions, e.g., privacy or ethical. The data presented in this study are available on request from the corresponding authors.
